# A Replicating Modified Vaccinia Tiantan Strain Expressing an Avian-Derived Influenza H5N1 Hemagglutinin Induce Broadly Neutralizing Antibodies and Cross-Clade Protective Immunity in Mice

**DOI:** 10.1371/journal.pone.0083274

**Published:** 2013-12-17

**Authors:** Haixia Xiao, Li Liu, Qingyu Zhu, Zhiwu Tan, Wenbo Yu, Xian Tang, Dawei Zhan, Yanhua Du, Haibo Wang, Di Liu, Zhixin Li, Kwok-Yung Yuen, David D. Ho, George F. Gao, Zhiwei Chen

**Affiliations:** 1 Laboratory of Protein Engineering and Vaccines, Tianjin Institute of Industrial Biotechnology, Chinese Academy of Sciences, Tianjin, China; 2 AIDS Institute, Li Ka Shing Faculty of Medicine, The University of Hong Kong, Hong Kong SAR, China; 3 CAS Key Laboratory of Pathogenic Microbiology and Immunology, Institute of Microbiology, Chinese Academy of Sciences, Beijing, China; 4 Department of Microbiology and Research Center of Infection and Immunology, Li Ka Shing Faculty of Medicine, The University of Hong Kong, Hong Kong SAR, China; 5 State Key Laboratory of Pathogens and Biosecurity, Institute of Microbiology and Epidemiology, Academy of Military Medical Sciences, Beijing, China; 6 Genomics Research Center, Academia Sinica, Taipei, Taiwan; 7 The Aaron Diamond AIDS Research Center, The Rockefeller University, New York, New York, United States of America, and the The University of Hong Kong, Hong Kong SAR, China; Shanghai Medical College, Fudan University, China

## Abstract

To combat the possibility of a zoonotic H5N1 pandemic in a timely fashion, it is necessary to develop a vaccine that would confer protection against homologous and heterologous human H5N1 influenza viruses. Using a replicating modified vaccinia virus Tian Tan strain (MVTT) as a vaccine vector, we constructed MVTT_HA-QH_ and MVTT_HA-AH_, which expresses the H5 gene of a goose-derived Qinghai strain A/Bar-headed Goose/Qinghai/1/2005 or human-derived Anhui Strain A/Anhui/1/2005. The immunogenicity profiles of both vaccine candidates were evaluated. Vaccination with MVTT_HA-QH_ induced a significant level of neutralizing antibodies (Nabs) against a homologous strain and a wide range of H5N1 pseudoviruses (clades 1, 2.1, 2.2, 2.3.2, and 2.3.4). Neutralization tests (NT) and Haemagglutination inhibition (HI) antibodies inhibit the live autologous virus as well as a homologous A/Xingjiang/1/2006 and a heterologous A/Vietnam/1194/2004, representing two human isolates from clade 2.2 and clade 1, respectively. Importantly, mice vaccinated with intranasal MVTT_HA-QH_ were completely protected from challenge with lethal dosages of A/Bar-headed Goose/Qinghai/1/2005 and the A/Viet Nam/1194/2004, respectively, but not control mice that received a mock MVTT_S_ vaccine. However, MVTT_HA-AH_ induced much lower levels of NT against its autologous strain. Our results suggest that it is feasible to use the H5 gene from A/Bar-headed Goose/Qinghai/1/2005 to construct an effective vaccine, when using MVTT as a vector, to prevent infections against homologous and genetically divergent human H5N1 influenza viruses.

## Introduction

Highly pathogenic avian influenza A virus (HPAIV) H5N1 has long been carried by wild aquatic bird populations, spread throughout the world via either the poultry transportation or the migratory bird flyway, and has killed or led to the culling of hundreds of millions of birds [1], [2]. H5N1 was shown to be lethal to human in 1997 when 6 of the 18 infected human cases in Hong Kong died [3]–[5]. Although H5N1 viruses have not yet been transmitted between humans, cross-species transmission of these viruses to human has been documented in 633 cases, with a mortality rate of 59.6%, since the reemergence of H5N1 viruses in 2003 [6]. Several recent independent studies suggested that H5N1 viruses might require very few amino acid substitutions to become transmissible via respiratory droplets between mammals [7]–[9]. Therefore, great concern has been raised in the ability of H5N1 viruses to efficiently spread between humans and become a pandemic threat, thus making an H5N1 influenza vaccine an integral part of any pandemic preparedness plan [10].

Broad cross-protection is a highly desirable feature of an H5N1 vaccine to avoid the possible pandemic of H5N1 influenza viruses. However, the efficacies of currently licensed vaccines appear to be insufficient partially due to the antigenic diversity present in the virus, restricting the utility of the vaccine to a small number of specific strains. Whilst the identity of any specific pandemic strain is rather difficult to predict before the event, it would take 4–6 months or more to deliver a vaccine using current manufacturing technologies [11], [12]. Therefore, great efforts have been made toward developing vaccines with broad cross-protection against H5N1 influenza viruses, as well as improving vaccine production methods to shorten the lead-time to vaccine delivery. New approaches, such as virus-like particles (VLPs), naked DNA and adenoviral and vaccinia vector-based vaccines have been developed to prevent H5N1 viral infections [10], [13], [14]. It was reported that inactivated H5N1 influenza viruses of clades 1 and 2.1 and virus-like particles (VLPs) containing the HA, NA and M1 proteins of IN5/05 and VN/1203 showed significant cross-protective potential [15]–[17]. Non-replicating vaccinia vectors, such as the Modified Vaccinia Virus Ankara (MVA) carrying an HA derived from the VN/1203 strain, displayed a high level of cross-protection [18]. A veterinary vaccine expressing the H5 gene of the A/Bar-headed Goose/Qinghai/1/2005 (A/BhG/QH/1/05) also provided protection against lethal challenges of homologous and heterologous avian H5N1 influenza viruses [19].

In our previous study, we generated a replicating Modified Vaccinia Tian Tan (MVTT) from Vaccinia Tian Tan (VTT) by removing the hemagglutinin gene and an 11,944bp genomic region from the HindIII fragment C2L to F3L in VTT [20]. VTT has been used extensively as a smallpox vaccine for millions of people in China before the 1980's [21] and was successfully developed into a vaccine vector for rabies and hepatitis B viruses [22], [23]. Compared to VTT, MVTT is very safe, as it has been shown to not replicate in mouse brain and does not cause death after intracranial injection or body weight loss after intranasal inoculation in immuno-deficient mice [20]. Moreover, using the spike glycoprotein (S) of SARS-CoV as the test antigen, we found that MVTT is superior to MVA for inducing high levels of neutralizing antibody via mucosal vaccination [24]. In this report, using MVTT as a live vaccine vector, we constructed MVTT_HA-QH_ and MVTT_HA-AH_, which expresses the H5 gene of a goose-derived Qinghai strain A/Bar-headed Goose/Qinghai/1/2005 or human-derived Anhui Strain A/Anhui/1/2005. We determined the immunogenicity and efficacy of both vaccine constructs in mice against homologous and heterologous human H5N1 influenza viruses.

## Materials and Methods

Our experimental protocols were approved by The Institutional Animal Care and Use Committee (IACUC) of the Chinese Academy of Military Medical Science, Beijing, China.

### Viruses and cells

HA^XJ^/WSN stands for the recombinant influenza A virus with its HA gene derived from the human strain A/Xingjiang/1/06 (H5N1) and the other seven genes from A/WSN/33 (H1N1). This reverse genetics system was kindly provided by Yoshiro Kawaoka [25]. Two wild-type H5N1 influenza viruses, A/BhG/QH/1/05 and A/Vietnam/1194/2004 (A/VN/1194/04), were used for challenge in this study. A/BhG/QH/1/05 and A/VN/1194/04 were cultivated in 10-day-old embryonated SPF chicken eggs (Beijing Merial Vital Laboratory Animal Technology Co., Ltd., Beijing, China), while A/HA^XJ^/WSN was propagated on Madin-Darby canine kidney (MDCK) cells. MDCK cells and Vero cells were cultured in Dulbecco's modified Eagle's minimum essential medium (DMEM) supplemented with 10% fetal calf serum (FCS) at 37°C in an atmosphere of 5% CO_2_. Allantoic fluids and cell cultures were harvested and stored at −70°C. The fifty percent mouse lethal dose (MLD_50_) of each strain was determined in 6-week-old female BALB/c mice and the fifty percent tissue culture infectious dose (TCID_50_) was titrated in MDCK cells. All of these experiments were conducted in a bio-safety level-3 laboratory. A group of diverse H5N1 pseudoviruses were prepared according to recently described methods [10].

### Construction of recombinant MVTT_HA-QH_ and MVTT_HA-AH_

The HA gene of A/BhG/QH/1/05 was amplified from the total RNA of the virus by RT-PCR. The HA gene of A/Anhui/1/2005 was kindly provided by Dr. David Ho and amplified by PCR. Each HA ORF gene was then inserted into a vaccinia shuttle vector pZC_XZ_ under the strong synthetic promoter pSYN and subsequently recombined into the promoter region of the MVTT HA gene to generate MVTT_HA-QH_ and MVTT_HA-AH_ using a homologous recombination method by transfection, as previously described [26]. In brief, Vero cells were infected with MVTT and subsequently transfected with a shuttle vector pZCxz containing the HA gene of influenza H5N1 flanked with MVTT HA sequences [26]. The recombinant virus was selected through six rounds of plaque purification under agar and confirmed by immunostaining assay using an anti-H5 serum [26]. The insertion of full length gene of H5N1 HA was further confirmed by PCR using specific primers. Total DNA was extracted from MVTT_HA-QH_, MVTT_HA-AH_ or MVTT_SIV_ infected Vero cells as described before [24]. The presence of full-length H5N1 *HA* genes were evidenced by the amplification of around 1700 bp fragments using the following pairs of primers: Anhui-F 5′-ATGGAGAAGATCGTGCTGCTG, and Anhui-B 5′-GATGCAGATTCTGCACTGCAGGCT; Qinghai-F 5′ ATGGAGAAAATAGTGCTTCT: Qinghai-B 5′ AATGCAAATTCTGCATTGTA, respectively. MVTT_HA-QH_ and MVTT_HA-AH_ viral stocks were propagated in Vero cells and then purified by ultracentrifugation through a 36% sucrose cushion. Both viral stocks were titrated simultaneously in Vero cells by a plaque forming assay using crystal violet staining or counting the plaques with GFP expression [26].

### Animal immunization

Six-week-old female BALB/c mice were used in all animal experiments. Three groups of mice (5 in each group) were immunized with 3.0×10^6^ plaque formation unit (PFU) of MVTT_HA-QH_ or MVTT_HA-AH_ through intranasal, intramuscular or oral routes at day 0 and day 31, respectively. The same number of mice was included in control groups given either PBS or MVTT_S_. MVTT_S_ is a recombinant virus expressing the spike glycoprotein of SARS-CoV, as we recently described [24]. Blood samples from each mouse were collected 2 weeks after each vaccination for measurement of antibody responses. Splenocytes were also collected for measuring cell-mediated immune response after stimulation with an H-2K^d^-restricted T-cell epitope IYSTVASSL, as previously described by others [27].

### Detection of immune responses

Sera from mice were treated with receptor-destroying enzyme (RDE, purchased from the Institute of Viral Disease Control and Prevention, Chinese Center for Disease Control and Prevention) in the ratio of 1∶3 and heat-inactivated for 30 minutes at 56°C. Neutralization tests (NT) using the diverse H5N1 pseudoviruses was performed according to recently described methods [10]. Haemagglutination inhibition (HI) tests were performed with 1% chicken red blood cells according to a method recommended by the World Organization for Animal Health (http://www.oie.int/Eng/Normes/Mmanual/A_00037.htm). Serum samples were also tested for neutralizing antibody against live A/BhG/QH1/05, A/HA^XJ^/WSN and A/VN/1194/04 by a traditional micro-neutralization assay. Briefly, heat-inactivated sera were first two-fold diluted with DMEM, and then mixed with an equal volume of 100 TCID_50_ of each of H5N1 influenza viruses. After an incubation period of 1 hour at 37°C, 200 μl of serum-virus mixtures were transferred onto MDCK cells in a 96-well plate, in triplicates. These cells were observed for any cytopathic effect (CPE), daily for 3 consecutive days. The titer of neutralizing antibody was defined as the highest serum dilution that inhibits the formation of 50% CPE. The cell-mediated immune response was determined by measuring H5-specific IFN-γ released by splenocytes according to a previously published ELIspot assay [10].

### Protection of mice against viral challenge

Mouse models have been commonly used for evaluating vaccines for influenza A (H5N1) viruses isolated from humans [28]. Groups of nine mice were either immunized with 1.5×10^6^ or 3.0×10^6^ PFU of MVTT_HA-QH_ and MVTT_S_, respectively, twice, with a one month interval. Immunized mice were challenged with either 100 MLD_50_ of A/BhG/QH/1/05 or 100 MLD_50_ of A/VN/1194/04 at 3 weeks post second immunization. Body weight change, number of deaths and clinical signs were closely monitored after viral challenge twice a day. The animals were humanely euthanized by intraperitoneal injection of pentobarbital sodium salt (100 mg/kg) and followed by cervical dislocation, when weight loss is greater than 20% body weight at the start of the experiment for 72 hours. Pentobarbital sodium salt was used to minimize suffering of the animals. The percentage of survival was calculated as previously described [10].

### Viral isolation

To measure viral replication *in vivo*, tissue samples (brain and lung) were taken from two mice in each group for viral isolation on the 3^rd^ and 6^th^ day post infection, respectively. After tissue samples were weighed and homogenized, 0.1 ml of each supernatant was inoculated into the allantoic cavity of 10 day-old embryonated SPF chicken eggs. The allantoic fluids were subsequently harvested and the HA activity was tested for the detection of virus replication.

### Statistical Analysis

Animal experiments to evaluate immune responses were repeated at least twice (*n* = 5 per group). The response of each mouse was counted as an individual data point for statistical analysis. Virus challenge studies were performed twice (*n* = 5 or 9 per group). Data obtained from animal studies and pseudotyped virus neutralization assays were examined by using one-way ANOVA. Differences were considered significant at *P*<0.05.

## Results

### Design and construction of MVTT viruses expressing H5 hemagglutinins

**Figure 1:**
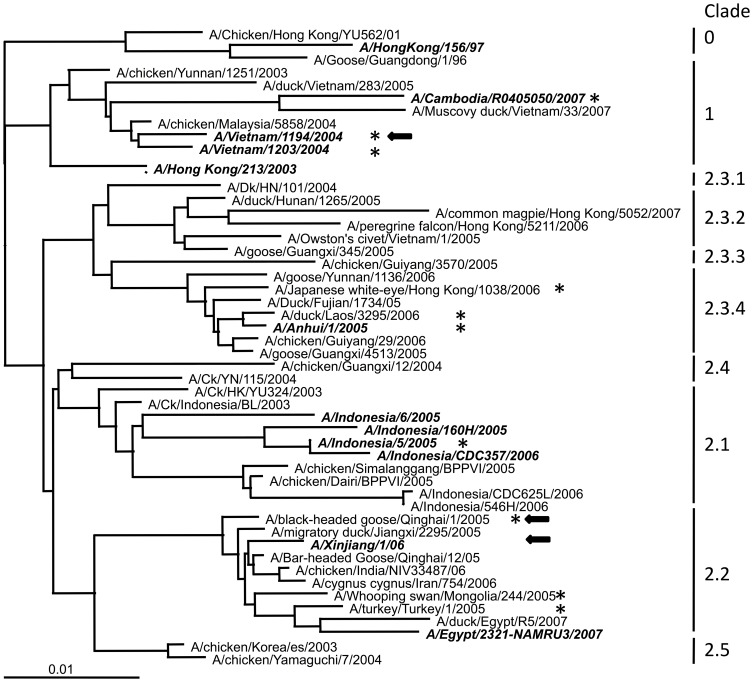
Phylogenetic relationship of the HA genes of influenza H5N1 viruses of multiple clades. This Neighbor-Joining tree is constructed using a computer program implemented in the ClustalX program (version 2.0) as previously described [42]. The clade classification is according to the reference sequences recommended by WHO. The asterisks highlight the vaccine strains recommended by WHO. Human isolates are italicized. The three live viruses that are included in this study are marked with arrowheads.

The HA gene of A/BhG/QH/1/05 and A/Anhui/1/2005 were chosen for vaccine construction because A/BhG/QH/1/05 represents the prototype of clade 2.2 highly pathogenic influenza A H5N1 viruses, which has led to sporadic human infections around the world (Fig. 1), whilst A/Anhui/1/2005 is a WHO recommended vaccine strain isolated from a human patient, which may induce a better immune response against human isolates. We analyzed the sequences of the HA genes derived from the human A/Xingjiang/1/06 strain and those from other countries. When compared with the HA of A/BhG/QH/1/05, the major antigenic sites within clade 2.2 were relatively conserved despite viral evolution occurring over two to three years outside the Qinghai Lake area (Table 1). A/Anhui/1/2005 belongs to clade 2.3.4, in which the major antigenic sites are very different from clade 2.2, but rather conserved within clade 2.3.4. We hypothesized that vaccines based on the HA would at least offer protection to human H5N1 influenza viruses of the same clade.

**Table 1:** Sequence variation of HA gene of H5N1 influenza viruses at the indicated antigenic sites.

		Amino acid residue at the indicated antigenic site and position																				
Clade	Virus	E		B	RBS	A		B	Gly				B	D		E		C				
		86	94	124	129	138	140	155	156	174	181	183	189	212	227	263	269	277	282	310		322
**2.2**	A/BHG/Qinghai/1/2005*	**A**	**N**	**D**	**S**	**Q**	**R**	**N**	**A**	**V**	**P**	**D**	**R**	**K**	**E**	**T**	**L**	**K**	**I**	**R**	**Q**	
**2.2**	***A/Xingjiang/1/06***	**V**	**.**	**.**	**.**	**.**	**.**	**.**	**T**	**.**	**.**	**.**	**.**	**.**	**.**	**.**	**.**	**.**	**.**	**.**	**.**	
2.2	*A/Egypt/2321NAMRU3/2007*	.	D	.	.	.	.	.	.	.	.	.	.	.	.	.	.	.	.	.	.	
2.2	A/turkey/Turkey/1/2005*	.	.	.	A	.	.	.	.	.	.	.	.	.	.	.	.	.	.	.	.	
2.2	A/WS/Mongolia/244/2005*	.	.	.	.	.	.	.	.	.	.	.	.	.	.	.	.	.	.	.	.	
2.1	A/Indonesia/5/2005*	T	S	.	.	L	S	S	T	.	.	.	.	.	.	A	.	.	M	.	.	
2.1	A/Chick/Indonesia/5/2004	.	.	.	.	.	K	S	T	.	.	.	.	.	.	A	.	.	M	.	.	
2.3.4	A/Anhui/1/2005*	.	.	.	.	.	T	.	T	I	S	.	K	.	D	A	V	.	.	K	L	
2.3.4	A/duck/Fujian/1734/2005	.	.	.	.	.	T	.	T	I	S	.	K	.	D	A	V	M	.	K	L	
2.3.4	A/duck/Laos/3295/2006*	.	.	.	.	.	T	.	T	I	S	.	K	.	D	A	V	.	.	K	L	
2.3.4	A/Jpn-WE/HK/1038/2006*	.	.	.	.	.	T	.	K	I	S	.	K	.	D	A	V	.	.	K	L	
2.3.2	A/duck/China/E319-2/03	.	.	.	.	.	S	D	.	.	.	.	.	.	D	A	V	.	M	K	.	
1	*A/Hong Kong/213/03*	.	D	S	L	.	K	.	.	.	.	.	.	.	.	A	.	.	M	.	.	
**1**	A/Vietnam/1194/2004*	**V**	**D**	**S**	**L**	**.**	**K**	**S**	**T**	**.**	**.**	**.**	**K**	**R**	**.**	**.**	**.**	**.**	**M**	**.**	**.**	
1	A/Vietnam/1203/2004*	V	D	S	L	.	K	S	T	.	.	.	K	R	.	.	.	.	M	.	.	
1	*A/Cambodia/R0405050/2007*	V	D	S	L	.	K	.	T	.	.	.	N	R	.	.	.	.	M	.	.	

Notes: The amino acid positions are based on mature H5 sequences. Three live viral strains that are included in this study are in bold.

Human isolates are in italic. The asterisk indicates a few WHO-recommended vaccine strains.

Abbreviations: RBS, receptor binding site; Gly, glycosylation site; BHG, bar-headed goose; Jpn-WE, Japanese white-eye.

**Figure 2:**
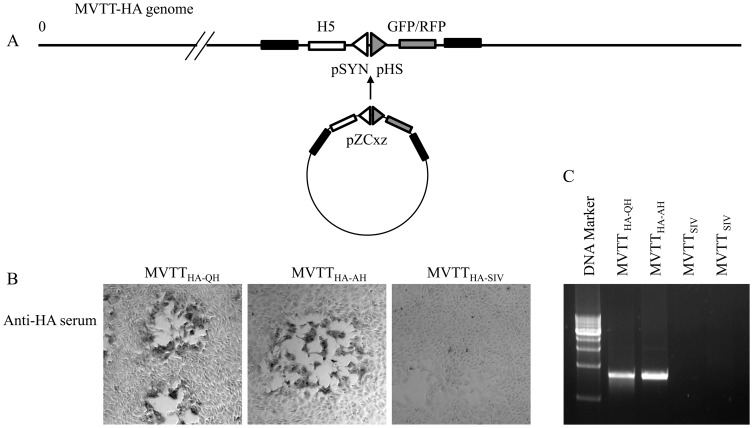
The schematic representation of MVTT_-HA-QH_ and MVTT_-HA-AH_ construction and the expression of the HA protein in cells infected with MVTT_-HA-QH_ or MVTT_-HA-AH_. (A) The HA gene was introduced, together with GFP gene or RFP gene, each under a separate promoter, into the genome of MVTT. The restriction enzymes Bam HI and Xho I were used for constructing MVTT_-HA-QH_ and MVTT_-HA-AH_. The insertion region corresponds to the Del III region of MVA. (B) The HA protein was detected on Vero cells infected with MVTT_-HA-QH_ or MVTT_-HA-AH_ using mouse anti-HA serum in an immunohistochemical staining assay. No HA protein expression was detected on Vero cells infected with the control virus MVTT_SIV_. (C) The full length HA gene was detected in the total DNA extracted from Vero cells infected MVTT_-HA-QH_ and MVTT_-HA-AH_ using specific primers for H5N1 Qinghai or Anhui strain. No H5 gene was detected in total DNA extracted from Vero cells infected control virus MVTT_ SIV_ using both primers.

A modified shuttle vector pZCxz was constructed to target the H5N1 HA gene into the MVTT genome in the location of the MVTT HA gene. This shuttle vector contains dual promoters that allow the simultaneous expression of two target genes. Within this vector, the HA gene of A/BhG/QH/1/05 or A/Anhui/1/2005 was constructed under the strong synthetic promoter pSYN, whereas a reporter GFP or RFP gene was under a separate relatively weaker promoter pH 5 (Fig.2A). Since both genes were included within the same insertion frame, the reporter gene served as a surrogate marker for the selection of recombinant VTT carrying the HA gene. Using this technique, we were able to generate and to purify the recombinant virus MVTT_HA-QH_ and MVTT_HA-AH_. The positive plaque was selected under a fluorescence microscope and subsequently confirmed by immunohistochemcal staining assay using a mouse anti-H5 serum (Fig. 2B). To further confirm that the full length H5N1 H5 gene was inserted into the MVTT vector, the total DNA was extracted from Vero cells infected by either MVTT_HA-QH_ or MVTT_HA-AH_. Total DNA was extracted from MVTT_SIV_ infected Vero cells as negative control. The full-length H5N1 *HA* genes were amplified from the total DNA extracted from both MVTT_HA-QH_ and MVTT_HA-AH_ infected cells using specific primers, but not MVTT_SIV_ infected Vero cells (Fig. 2C). These results confirmed that we have successful constructed MVTT virus expressing H5 gene from A/BhG/QH/1/05 and A/Anhui/1/2005 strains, respectively.

### MVTT_HA-QH_ induced specific immune responses against homologous H5N1 influenza virus

**Figure 3:**
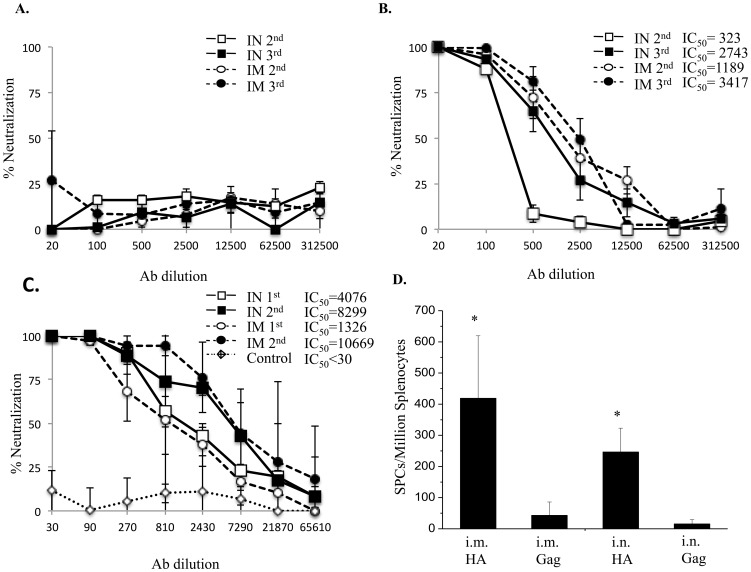
HA-specific Nab and IFN-γ-secreting CD8^+^ T-cell responses to MVTT_HA-QH_ or MVTT_HA-AH_ vaccinations. BALB/c mice were vaccinated three times or twice with MVTT_HA-QH_ (A) or MVTT_HA-AH_ (B, C), respectively, at three-week intervals via intranasal (I.N.) and intramuscular (I.M.) inoculations, respectively. The antiserum was collected at two weeks after each vaccination for analysis of HA-specific Nabs against H5N1 Qinghai strain (**A, C**) or Anhui strain (**B**), respectively. Control animals were given MVTT_S_ via the same corresponding routes. (**D**) Splenocytes of immunized mice were obtained after two immunizations and assessed by an ELIspot assay using a specific peptide (HA 9mer) or an irrelevant peptide (HIV Gag 9mer). *P*<0.01, MVTT_HA-QH_ vs. MVTT_S_.

To determine the immunogenicity of MVTT_HA-QH_ and MVTT_HA-AH_, two groups of mice were immunized twice with 3×10^6^ PFU of recombinant virus on day 0 and day 31, via the intranasal (I.N.) and intramuscular (I.M.) routes, respectively. Serum samples collected on day 0 and day 28 after the first immunization and 2 weeks after the second inoculation (day 45) were subjected to a neutralization (NT) assay by using a previously described pseudoviral assay [10]. We found that MVTT_HA-QH_ was able to induce potent NT response against the autologous viral strain with a dilution factor of IC_50_ over 1000 after the first immunization (Fig. 3A). The I.N. route induced seemingly higher levels of NT responses than the I.M. route after the first immunization (IC_50_ titer: 4076 vs. 1326). Moreover, the second immunization boosted primary responses by 2 (I.N.) to 8 (I.M.) fold for NT titers. Mice who received the control MVTT_SIV_ did not generate any responses, as expected (Fig. 3A). In contrast, after the 2^nd^ immunization, none of the mice immunized with MVTT_HA-AH_ was able to induce any NT response against its homologous strain (Fig. 3B). We, therefore, conducted a 3^rd^ immunization with the same dose of MVTT_HA-AH_ three weeks after the 2^nd^ immunization, but only one in five mice was able to produce NT responses against its homologous strain (IC_50_ titer: 70). However, MVTT_HA-AH_ was able to induce potent NT responses with a dilution factor of IC_50_ around 1000 after the 2nd immunization against the Qinghai strain (Fig. 3C). Furthermore, compared to MVTT_HA-QH_, MVTT_HA-AH_ induced seemingly lower levels of NT responses through the I.N. route than through the I.M. route after the 2nd immunization. The 3rd immunization boosted the primary responses for NT titers. These results suggest that immunization with MVTT_HA-AH_ was successful, however, A/Anhui/1/2005 is likely to be less sensitive to the neutralizing activity of vaccine-induced antibody. Moreover, HA from A/Anhui/1/2005 is less antigenic compared to HA from the Qinghai strain.

We then chose MVTT-_HA-QH_ for the evaluation of cell mediated H5-specific responses. We found that both routes of vaccination were able to induce H5-specifc IFN-γ releasing CD8^+^ T-cells by measuring the spot forming counts (SFC) using ELIspot assay (Fig. 3D). On average, about 420 SFC (I.M.) and 250 SFC (I.N.) were detected among one million splenocytes. Thus, MVTT_HA-QH_ was found to be immunogenic in mice for inducing both antibody and cellular immune responses and a better vaccine candidate for the prevention of the H5N1 pandemic compared to MVTT_HA-AH_. We, therefore, chose MVTT-_HA-QH_ for further evaluation.

### Neutralizing antibodies induced by MVTT_HA-QH_ inhibited a wide range of divergent H5N1 influenza pseudoviruses

**Figure 4:**
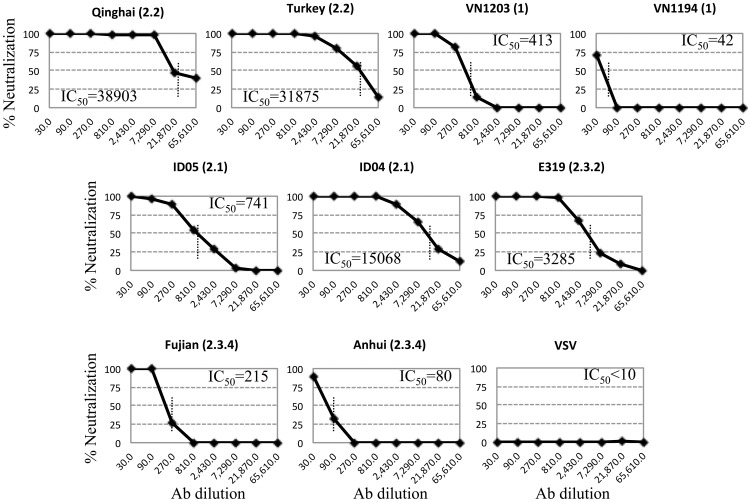
Cross-neutralization of the infectivity of HA-pseudotyped viruses by antisera obtained from mice after receiving two injections of MVTT_HA-QH_. The IC_50_ is defined as the mean reciprocal of the antiserum dilution at which virus entry is 50% inhibited (dashed line). Data were collected from three independent experiments and the mean values are presented.

To further determine the cross-clade NT activities of MVTT_HA-QH_, we tested the immune sera against a group of divergent H5N1 influenza pseudoviruses. The group included viruses from clades 1 (VN1203 and VN1194), 2.1 (ID05 and ID04), 2.2 (Qinghai and Turkey), 2.3.2 (Chinese duck E319) and 2.3.4 (Fujian and Anhui) (Fig. 1 and Table 1). Although the levels of cross-neutralization varied, the Nabs induced by MVTT_HA-QH_ were able to neutralize pseudoviruses from all major clades tested (Fig. 4). Those viruses from clade 2.2 were most optimally inhibited, followed by clades 2.1, 2.3.2, 2.3.4 and 1, which is consistent to the amount of sequence variation at the antigenic sites (Table 1).

### MVTT_HA-QH_ induced potent antibody response and protected mice against live homologous H5N1 influenza virus

To further determine the efficacy of vaccination, three groups of mice were immunized twice with 3×10^6^ PFU of MVTT_HA-QH_ on day 0 and 31, via the intranasal, intramuscular and oral administration routes, respectively. We included the oral route, as it is one of the easiest methods of vaccination for resource-limited settings. Serum samples collected on day 0 before vaccination, day 28 after the first immunization and 2 weeks after the second inoculation (day 45) were subjected to HI and NT assays using the live homologous A/BhG/QH/1/05 strain. We found that all three vaccination routes were able to induce both HI and NT responses (Table 2). The I.N. route induced seemingly higher levels of both HI and NT responses than the other two routes after the first immunization. Moreover, the second immunization boosted the primary responses significantly, more specifically, 4–16 fold for HI and 5–7 fold for NT titers, which are consistent to findings using the pseudoviral neutralization assay (Fig. 3A). Unexpectedly, two of the five mice in the oral group did not respond to the vaccine after two immunizations for unknown reasons. For the control, mice receiving PBS did not generate any responses, as expected. We did not conduct the ELIspot assay in this experiment because the animals were kept for subsequent viral challenge.

**Table 2:** The antibody level of immunized mice via HI and NT experiments.

		0 dpv†		28 dpv		45 dpv	
Groups*	Dose (pfu)	HI	NT	HI	NT	HI	NT
PBS (i.n.)	N/A	0	0	0	0	0	0
rVTT-HA(i.m.)	3×10^6^	0	0	2^3^	120.7	2^7^	623.25
rVTT-HA (i.n.)	3×10^6^	0	0	2^5^	163	2^7^	1112.5
rVTT-HA (oral)‡	3×10^6^	0	0	0, 2^3^	0, <40	0, 2^8^	0, 1778

*, i.n.: intranasal. i.m.: intramuscular.

† dpv: day post primary vaccination.

‡ the separated values represent different antibody levels were detected. N/A: not available.

**Figure 5:**
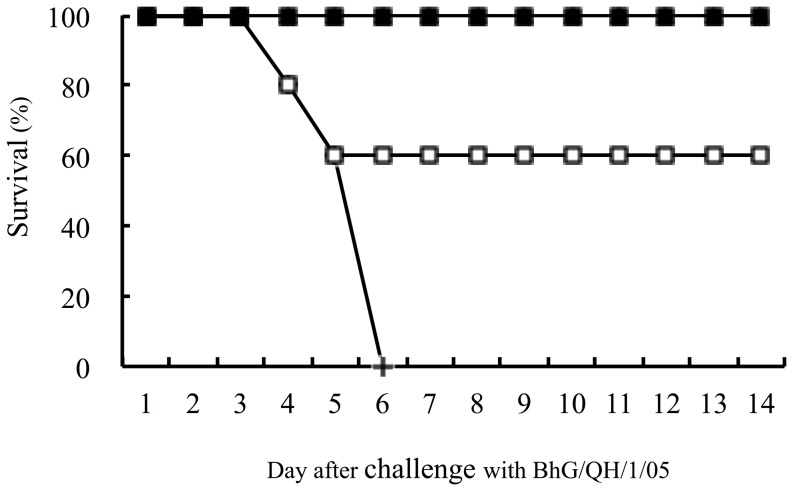
Vaccine protection against lethal challenge of pathogenic A/BhG/QH/1/05 virus. BALB/c mice were vaccinated twice with MVTT_HA-QH_ via intranasal, oral and intramuscular routes, respectively. The vaccinated animals were challenged with 100 MLD_50_ A/BhG/QH/1/05 three weeks after the second immunization. Mice who received PBS were used as controls.

To determine whether the vaccine would offer any protection, vaccinated animals were challenged with the homologous pathogenic A/BhG/QH/1/05 virus strain three weeks after the second immunization. By evaluating the body weight change and the survival rate, we found that all animals with detectable antibody responses were completely protected (Fig. 5). Moreover, the protected mice exhibited no clinical signs of infection, including huddling, shivering and ruffled fur. In contrast, animals who received PBS and the two mice (2/5) that did not show any detectable antibody responses in the oral group, showed significant clinical signs of infection and died within 6 days post viral challenge.

### MVTT_HA-QH_ induced potent antibody response and protected mice against live heterologous human H5N1 influenza viruses

To determine whether or not MVTT_HA-QH_ would confer protection against heterologous human H5N1 influenza viruses, we immunized another four groups of mice via the I.N. and I.M. routes. Here, we included the mock vaccine MVTT_S_ for determination of whether the vaccine vector would induce any non-specific protection effects. Using the same vaccination protocol, but with a reduced dosage (1.5×10^6^ PFU of MVTT_HA-QH_), MVTT_HA-QH_ was able to induce both HI and NT responses, as expected (Table 3). In addition to the homologous virus, two heterologous human viruses, A/VN/1194/04 and A/HA^XJ^/WSN, were included for testing. Consistent to antigenic analysis (Table 1), similar levels of HI and NT responses were detected against A/HA^XJ^/WSN when compared with the homologous virus (Table 3). Despite antigenic variation, HI and NT responses were also detected against the clade 1 A/VN/1194/04 (Fig. 1 and Table 3). As expected, no H5-specific HI and NT responses were detected among mice immunized with MVTT_S_.

**Table 3:** Antibody titer against homologous and heterologous H5N1 influenza viruses.

	BhG/QH/1/05		VN/1194/04		HA^XJ^/WSN	
	HI*	NT	HI	NT	HI	NT
rVTT-HA (i.n.)	2^6^	501.5	2^3^	316	2^6^	668.8
rVTT-HA (i.m.)	2^7^	819.6	2^4^	425	2^7^	914.5
rVTT-S (i.n.)	0	0	0	0	0	0
rVTT-S (i.m.)	0	0	0	0	0	0

*Serum samples were collected and tested two weeks after the second vaccination.

**Figure 6:**
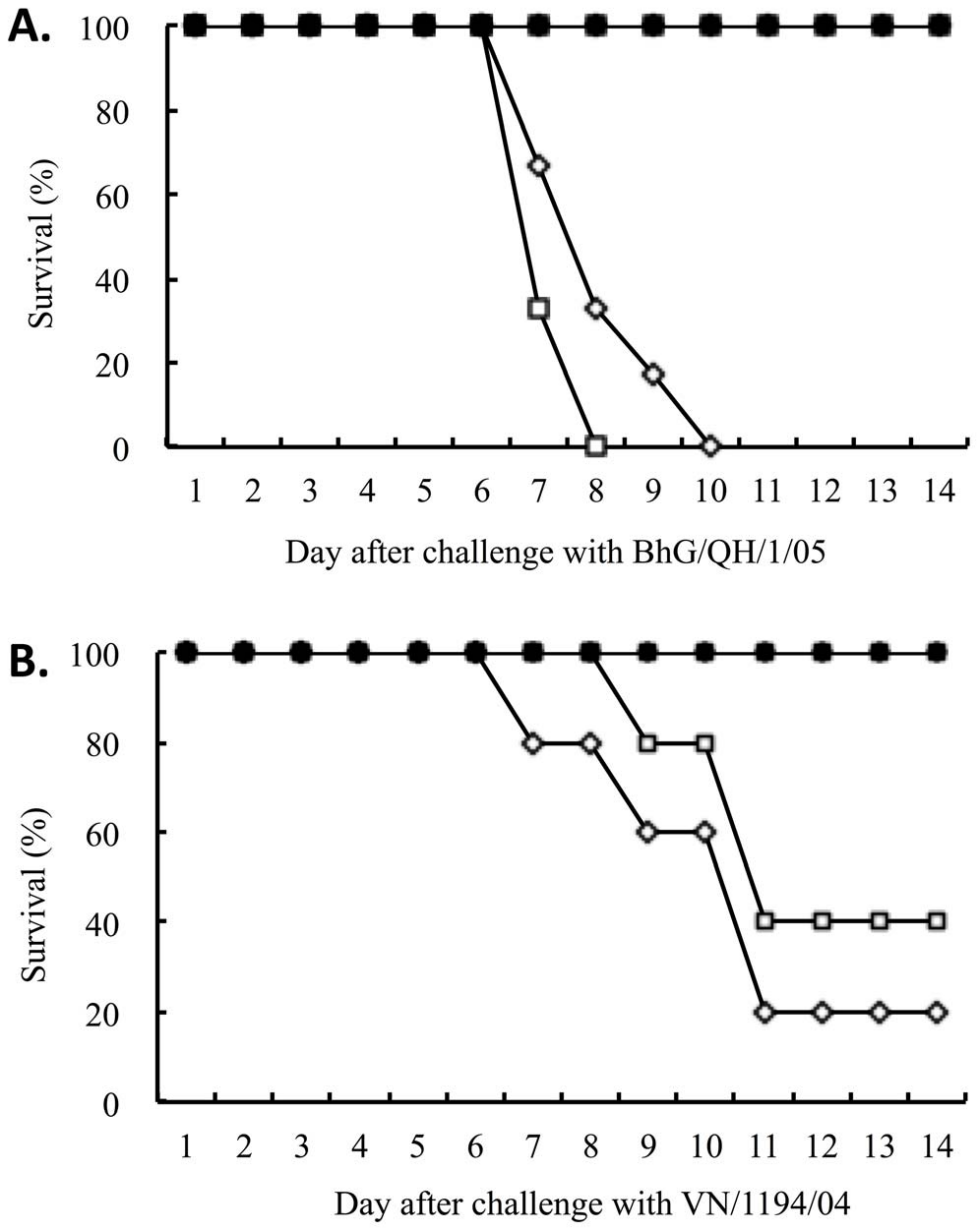
Vaccine protection against lethal challenges of homologous A/BhG/QH/1/05 (A) and heterologous A/VN/1194/04 (B) viruses. BALB/c mice were vaccinated twice with MVTT_HA-QH_ via intranasal and intramuscular routes, respectively. The vaccinated animals were challenged with 100 MLD_50_ of either A/BhG/QH/1/05 or A/VN/1194/04 three weeks after the second immunization. Mice who received MVTT_S_ were included as controls.

To further determine the efficacy of MVTT_HA-QH_, the vaccinated animals were challenged with pathogenic A/BhG/QH/1/05 and A/VN/1194/04 viruses 3 weeks post second immunization. Since A/HA^XJ^/WSN is not a pathogenic virus, it was not included in the challenge experiment. Consistent to our previous findings (Fig. 5), all animals with detectable antibody responses were completely protected against the homologous A/BhG/QH/1/05 challenge (Fig. 6A). Again, the protected mice exhibited no clinical signs of infection. Importantly, all animals with detectable antibody responses were also completely protected against the heterologous A/VN/1194/04 challenge (Fig. 6B). In contrast, animals which received MVTT_S_ showed significant clinical signs, including huddling, shivering, ruffled fur and body weight loss, and most of them died within 6 days post viral challenge. Despite the occurrence of clinical signs, three control animals (one in the I.M. group and two in the I.N. group) survived the A/VN/1194/04 challenge for unknown reasons (Fig. 6B).

To determine whether the MVTT_HA-QH_-induced immune responses could prevent viral replication *in vivo,* two mice from each group were sacrificed on days 3 and 6 post challenge with A/BhG/QH/1/05 and A/VN/1194/04, respectively. Samples from the lung and brain were then subjected to viral isolation. In agreement with the data shown in Fig. 5, no viruses were obtained from mice vaccinated with MVTT_HA-QH_ (Table 4). In contrast, viruses were readily isolated from mice who received MVTT_S_. Interestingly, on day 3 post viral challenge, A/BhG/QH/1/05 was easily detected, whereas A/VN/1194/04 was not, suggesting the higher infectivity and likely higher pathogenicity of the former virus in the murine model.

**Table 4:** Viral isolation in mice post lethal challenge with BhG/QH/1/05 and VN/1194/04.

	BhG/QH/1/05		VN/1194/04	
	3 dpi*	6 dpi	3 dpi	6 dpi.
MVTT-HA (i.n.)	0/2**	0/2	0/2	0/2
MVTT-HA (i.m.)	0/2	0/2	0/2	0/2
MVTT-S (i.n.)	2/2	2/2	0/2	2/2
MVTT-S (i.m.)	2/2	2/2	0/2	1/2

*, dpi: day post infection.

**, Two mice from each group were sacrificed for viral isolation at the indicated time points.

The fractions in the cells present the number of mouse (mice) with virus isolated/the number of mice from which tissue samples were collected.

## Discussion

In this study, we investigated the breath and efficacy of two MVTT based vaccines expressing H5 derived from A/Bar-headed Goose/Qinghai/1/2005 or A/Anhui/1/2005 against homologous and heterologous human H5N1 influenza viruses. HPAIV has been considered a serious public health threat with pandemic potential. If an HPAIV pandemic occurs, it is estimated to claim the lives of over a hundred million people [29], [30]. However, currently licensed vaccines are only effective to a small number of specific strains. The difficulty to predict the pandemic strain together with long and slow process of vaccine production make the efficient development of a vaccine with broad cross-protection an important step for pandemic preparedness. Here, we describe a Modified Vaccinia Virus TianTan strain (MVTT) based H5N1 vaccine, expressing the H5 gene of a goose-derived Qinghai strain A/Bar-headed Goose/Qinghai/1/2005, which can induce broadly neutralizing antibodies and cross-clade protective immunity in mice.

Currently, human vaccines have mainly focused on clade 1 strains, among which VNM/1203/04-based vaccines are the most extensively studied. In contrast, both A/BhG/QH/1/05 and A/Anhui/1/2005, although recommended by the WHO (World Health Organization) as one of the vaccine targeted strains, have yet to be comprehensively studied for their potential as a human vaccine candidate. In the past few years, viruses that are closely related to A/BhG/QH/1/05 have been found to have spread rapidly to Europe, India and Africa via migratory birds, and were likely the causative agents for several human infections throughout Europe and China, including our recently identified A/Xinjiang/1/06 [31]–[33]. In this study, we characterized the immunogenicity of MVTT_HA-QH_ and MVTT_HA-AH_, and the antibody response induced against multiple H5N1 influenza viruses representing clades 1 (VN1203 and VN1194), 2.1 (ID05 and ID04), 2.2 (Qinghai and Turkey), 2.3.2 (Chinese duck E319) and 2.3.4 (Fujian and Anhui) (Fig. 1) using the pseudoviral NT assay. We found that Nabs induced by MVTT_HA-QH_ neutralized pseudoviruses from all major clades tested. The MVTT_HA-QH_-induced serum inhibited viruses from clade 2.2 most optimally, followed by clades 2.1, 2.3.2, 2.3.4 and 1, suggesting that sequence variation in the antigenic sites probably accounted for the reduced NT titers of divergent H5N1 strains (Table 1). Moreover, we characterized a MVTT_HA-QH_ -induced antibody response against the human A/Xinjiang/1/06 strain (Fig. 1), a virus recently identified from a Chinese patient in Xinjiang province that is northwest to the Qinghai province. Despite sequence variations in the antigenic sites, probably due to adaptive infections in humans (Table 1), the HA gene of A/Xinjiang/1/06 remains closely related to A/BhG/QH/1/05 (Fig. 1). It is, therefore, not surprising to see that MVTT_HA-QH_ induced neutralizing and HI antibody responses that reacted equally well against both A/BhG/QH/1/05 and HA^XJ^/WSN (Table 3). In particular, the A86V and A156T mutations in A/Xinjiang/1/06 did not seem to confer resistance to the NT and HI antibodies induced by MVTT_HA-QH_ (Table 1). Furthermore, we determined the cross-reactivity of MVTT_HA-QH_–induced NT and HI antibodies against another human virus, the clade 1 A/VN/1194/04. Interestingly, although ten major antigenic mutations were identified between A/BhG/QH/1/05 and A/VN/1194/04 (Table 1), which accounted for titer drops in HI (∼8-fold) and live viral NT (∼2-fold) assays (Table 3), MVTT_HA-QH_ was still able to induce significant levels of NT and HI antibody responses against the heterologous human A/VN/1194/04 strain. Importantly, all MVTT_HA-QH_-vaccinations were completely protected against challenges with pathogenic A/BhG/QH/1/05 and A/VN/1194/04, respectively (Fig. 5 and 6). These findings indicated that MVTT_HA-QH_ displayed a potential to overcome the problem of antigenic drift between clade 1 and clade 2.2, and therefore, is an attractive target vaccine for further clinical development. H5 from A/Anhui/2/05 appeared to be a weak antigen when using MVTT as a vector. This was most likely caused by mutations inducing conformational changes and hid of antigenic sites of HA (Table. 1), because the HA protein was efficiently expressed in MVTT_-HA-AH_ infected Vero cells (Fig. 2), and MVTT_HA-AH_ was able to induce NT responses after the 2nd immunization against the Qinghai strain (Fig. 3C). Consistently, A/Anhui/1/2005 is less sensitive to the neutralizing activity of MVTT_-HA-QH_-induced antibodies compared to other H5N1 influenza viruses, including strain A/duck/Fujian/1734/2005, which also belongs to clade 2.3.4 (Fig. 3 and 4). Two out of five mice in the oral administration group did not respond to the MVTT_-HA-QH_ vaccine after two immunizations due to individual variance.

MVTT-based vaccines offer certain advantages over conventional influenza vaccines. With recent technical improvements, the time required for constructing MVTT-based vaccines is becoming significantly shortened. In comparison to adeno-vectored or DNA-based vaccines, vaccinia delivers a high level of foreign gene expression directly without time-consuming gene-optimization procedures [10], [13]. Critically, MVTT_HA-QH_ does not depend on SPF eggs or primary chicken embryo fibroblast [34], [35]. MVTT_HA-QH_ can be produced using WHO-recommended Vero cells with ease and without the requirement of bio-safety level-3 containment, which is necessary for inactivated vaccines. The large-scale production of MVTT_HA-QH_ is, therefore, feasible. Given the large capacity for housing large foreign gene inserts, it is possible to use MVTT to generate a multivalent-H5 vaccine in order to further improve the breath of cross-clade NT and HI antibody responses. When compared to the widely-used modified vaccinia Ankara (MVA) vector [36]–[41], we recently demonstrated that MVTT is superior to MVA for inducing high levels of systemic neutralizing antibodies against SARS coronavirus, especially through mucosal routes of vaccination [24]. To this end, we found that intranasal vaccination with MVTT_HA-QH_ induced consistently high levels of systemic NT and HI antibody responses that are equivalent to the outcomes of intramuscular injection. Moreover, intranasal vaccination can overcome pre-existing anti-vector immune responses, which offers advantages for the elderly who previously have received VTT against smallpox [24]. As a non-invasive procedure, intranasal vaccination also has great implications for mass vaccination of human populations, especially in developing countries. Lastly, intranasal vaccination has made it possible to develop MVTT_HA-QH_ as a potential aerosol veterinary vaccine, which, however, would require efficacy testing after being sprayed onto poultry and migratory bird populations. Given these reasons, our findings from this study have important implications in the fight against possible zoonotic HPAIV outbreaks.
