# Correction: A Replicating Modified Vaccinia Tiantan Strain Expressing an Avian-Derived Influenza H5N1 Hemagglutinin Induce Broadly Neutralizing Antibodies and Cross-Clade Protective Immunity in Mice

**DOI:** 10.1371/journal.pone.0115925

**Published:** 2014-12-12

**Authors:** The PLOS ONE Staff

There is an error in the penultimate sentence of the second paragraph in the “MVTT_HA-QH_ induced potent antibody response and protected mice against live heterologous human H5N1 influenza viruses” section of the Results. The correct sentence is: In contrast, animals which received MVTT_S_ showed significant clinical signs, including huddling, shivering, ruffled fur and body weight loss, and most of them died within 11 days post viral challenge.

There is an error in the second sentence of the third paragraph in the “MVTT_HA-QH_ induced potent antibody response and protected mice against live heterologous human H5N1 influenza viruses” section of the Results. The correct sentence is: Samples from the lung and brain were then subjected to viral isolation. In agreement with the data shown in Fig. 6, no viruses were obtained from mice vaccinated with MVTT_HA-QH_ (Table 4).

**Figure 6:**
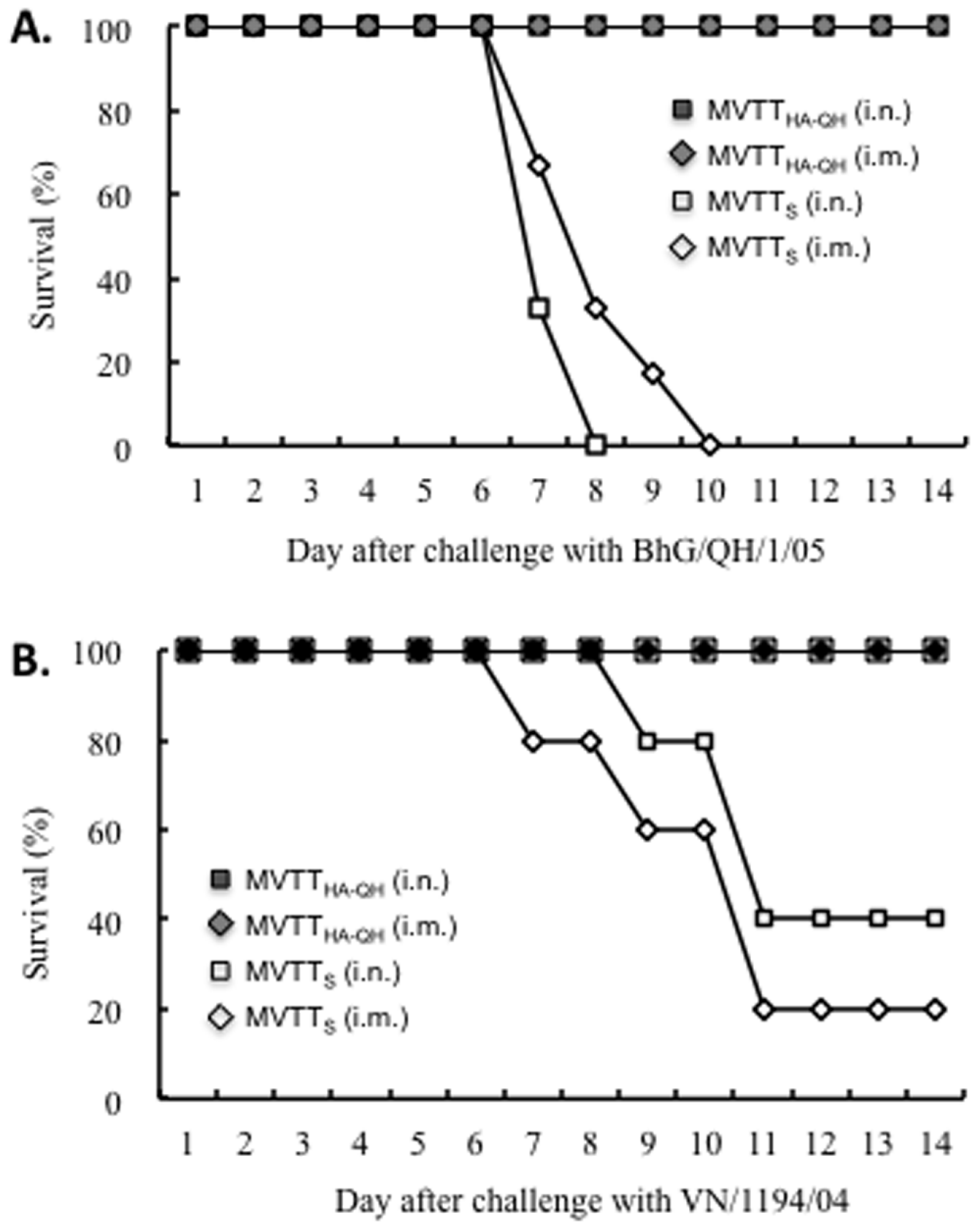
Vaccine protection against lethal challenges of homologous A/BhG/QH/1/05 (A) and heterologous A/VN/1194/04 (B) viruses. BALB/c mice were vaccinated twice with MVTT_HA-QH_ via intranasal and intramuscular routes, respectively. The vaccinated animals were challenged with 100 MLD_50_ of either A/BhG/QH/1/05 or A/VN/1194/04 three weeks after the second immunization. Mice who received MVTT_S_ were included as controls.

Figures 3, 5 and 6 are incorrect. Please see the corrected figures here.

**Figure 3:**
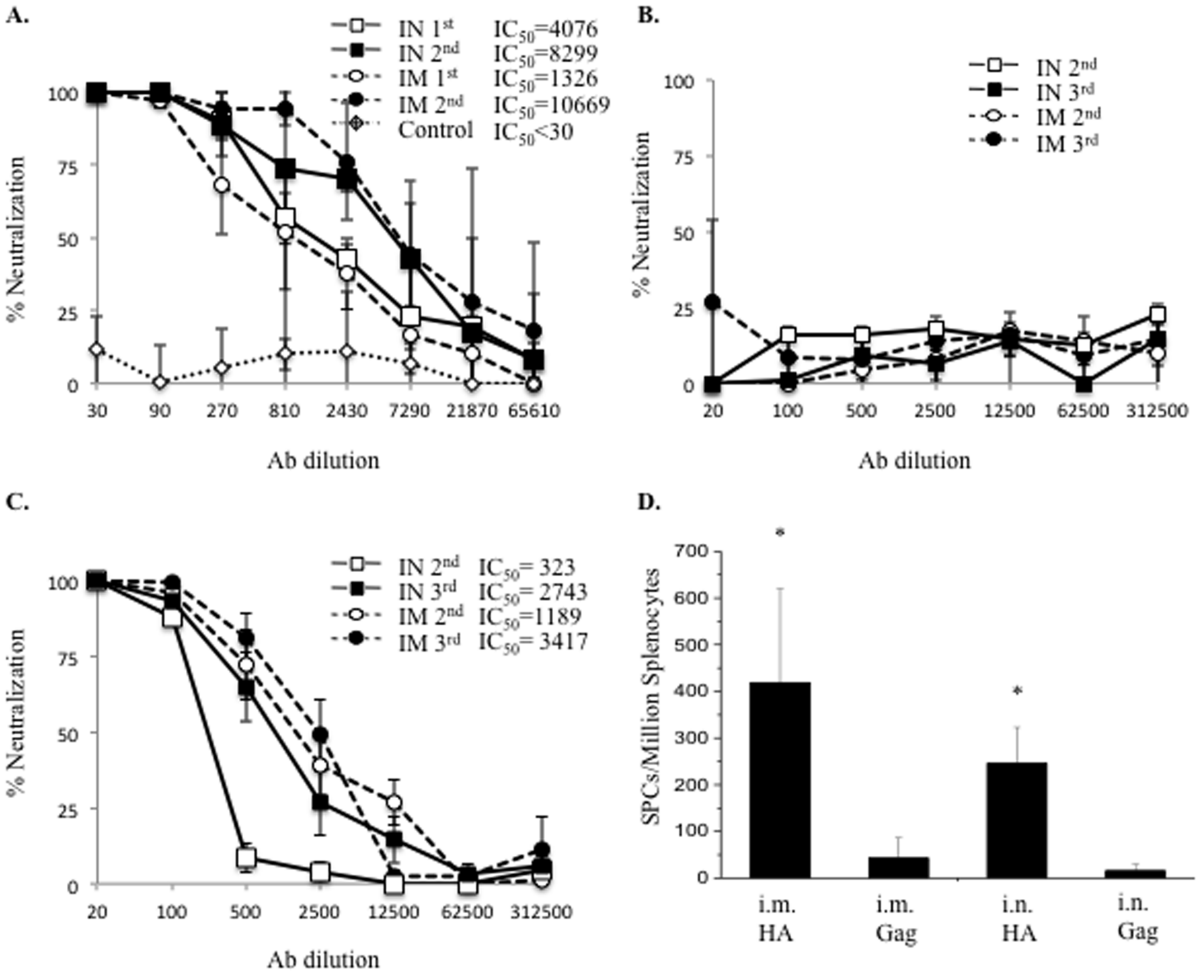
HA-specific Nab and IFN-γ-secreting CD8^+^ T-cell responses to MVTT_HA-QH_ or MVTT_HA-AH_ vaccinations. BALB/c mice were vaccinated three times or twice with MVTT_HA-QH_ (A) or MVTT_HA-AH_ (B, C), respectively, at three-week intervals via intranasal (I.N.) and intramuscular (I.M.) inoculations, respectively. The antiserum was collected at two weeks after each vaccination for analysis of HA-specific Nabs against H5N1 Qinghai strain (A, C) or Anhui strain (B), respectively. Control animals were given MVTT_S_ via the same corresponding routes. (D) Splenocytes of immunized mice were obtained after two immunizations and assessed by an ELIspot assay using a specific peptide (HA 9mer) or an irrelevant peptide (HIV Gag 9mer). P<0.01, MVTT_HA-QH_ vs. MVTT_S_.

**Figure 5:**
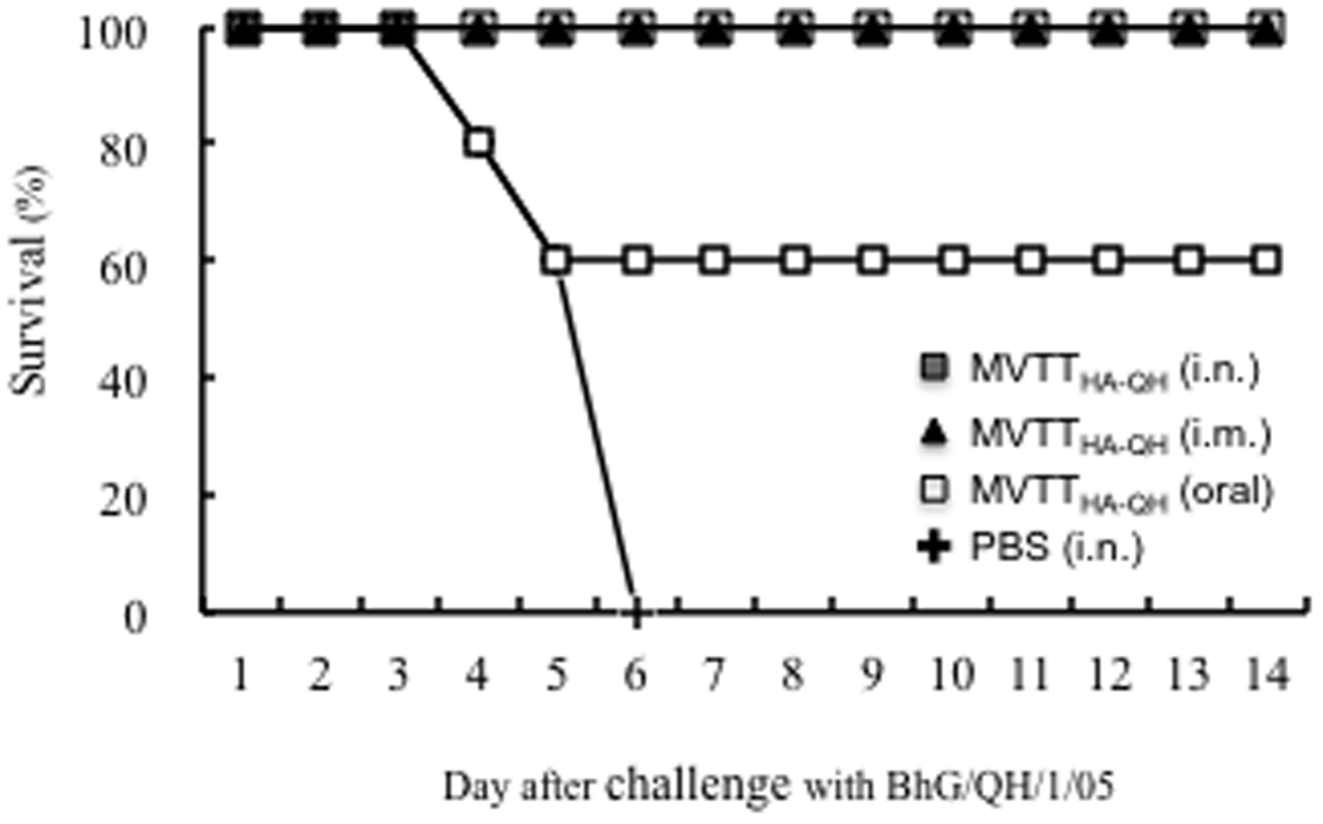
Vaccine protection against lethal challenge of pathogenic A/BhG/QH/1/05 virus. BALB/c mice were vaccinated twice with MVTT_HA-QH_ via intranasal, oral and intramuscular routes, respectively. The vaccinated animals were challenged with 100 MLD_50_ A/BhG/QH/1/05 three weeks after the second immunization. Mice who received PBS were used as controls.
